# Characterization and assessment of hydrogen leakage mechanisms in salt caverns

**DOI:** 10.1038/s41598-024-84505-x

**Published:** 2025-01-02

**Authors:** Mojtaba Ghaedi, Raoof Gholami

**Affiliations:** Department of Energy Resources, University of Stavanger, Stavanger, Norway

**Keywords:** Hydrogen storage, Salt cavern, Leakage, Numerical simulation, Diffusion, Viscous, Underground hydrogen storage, Fluid dynamics

## Abstract

Salt caverns are widely regarded as a suitable option for the underground storage of hydrogen. However, an accurate assessment of the hydrogen leakage through the walls of salt caverns into the surrounding formations remains crucial. In this work, the flow of hydrogen into the surrounding formation is evaluated by assuming that salt rock consists of bundles of tortuous nano-capillary tubes. A formulation was then proposed to model the flow in linear and radial domains. The formulations are based on a newly proposed unified gas flow model that is valid for the entire range of Knudsen numbers and accounts for gas slippage, bulk diffusion, and Knudsen diffusion. A finite-difference approximation with an iterative procedure was then used to treat the nonlinearity and solve the presented formulations. The formulations were validated against the experimental data reported in the literature. The results obtained indicated that for hydrogen flow over a wide range of pore radii and operating pressures and temperatures, the slippage flow regime must be considered. In a salt cavern with relevant dimensions and operating conditions, the cumulative hydrogen leakage after 30 years of cyclic storage was only 0.36% of the maximum storage capacity. It was also noticed that most of the leaked hydrogen would flow back into the salt cavern at times when the pressure in the salt cavern is lower than the surrounding pressure, e.g. during production and subsequent idle times. At low storage pressure and very tight salt rock, diffusion was the most important mechanism for hydrogen transport. At a high pressure though, viscous flow became the predominant leakage mechanism. The presence of a thin interlayer such as mudstone, carbonate, and anhydrite in the body of the salt rock can have a significant impact on the amount of leakage. It appeared that although increasing the maximum operating pressure from 120 to 135 bar only led to an 11.9% increase in the maximum storage capacity, the hydrogen loss increased significantly from 0.007% at 120 bar to 0.36% at 135 bar. Furthermore, although the absolute leakage rate for natural gas storage was higher than that for hydrogen storage, the relative leakage rate in relation to the maximum salt cavern capacity was much lower. The leakage range was also lower for natural gas storage compared to hydrogen storage. The formulations presented and the results obtained in this study can help to have a better understanding of the salt caverns when it comes to large-scale hydrogen storage.

## Introduction

In recent years, hydrogen (H_2_) has received significant attention as a clean energy carrier and an effective means of achieving net-zero emissions targets^1–3^. However, given the intermittency of renewable resources used to produce H_2_, cost-effective storage options are needed to maintain smart grid stability and balance fluctuations in energy demand. Underground storage sites such as salt caverns, depleted gas reservoirs, and saline aquifers, are among the suitable options for H_2_ storage, given their large-scale storage capacity^4–8^. Among these storage options, salt caverns are perhaps the best candidates due to several advantages: (i) they require less cushion gas compared to other storage options, (ii) have a very good sealing capacity given their tight structure, and (iii) may not trigger any interactions with H_2_ over time. Furthermore, salts are ductile formations and offer huge flexibility for multiple cycles of injection and withdrawal^7,9,10^. The caverns can also be constructed at shallower depths of 300 m to 2000 m through solution mining and offer a storage capacity of 5000 to 1,000,000 cubic meters^11^.

Salt caverns have long been used for the underground storage of natural gas. However, there is little experience with salt caverns when it comes to H_2_ storage. Pure H_2_ storage in salt caverns (> 95%) has been carried out in Clemens, Moss Bluff, and Spindletop in the USA, and in Teesside in the UK. The salt caverns at Teesside have been used for H_2_ storage since 1972, while H_2_ storage at Clemens Dome and Moss Bluff began in 1983 and 2007, respectively. This indicates the technical feasibility of H_2_ storage in salt caverns^7,9,12^.

H₂ losses during storage in salt caverns can be important from an economic and safety perspective. Wang et al.^13^ investigated the flow of various gases in bedded salt rock using experimental and numerical methods, considering diffusion and viscous flow mechanisms. They analyzed the effects of the different gases and found that H_2_ has a higher leakage compared to methane (CH_4_) and helium (He). They reported a leakage ratio of 5.76% (the cumulative standard volume of leaked H_2_ relative to the storage capacity) in the Jintan salt cavern in China over 30 years of storage operation, which is higher than the values reported in previous studies^14,15^. Chen et al.^14^ formulated the governing equations for one-dimensional and plane radial flow during gas seepage in the interbedded structure of bedded rock salt. Their analysis showed that the gas flow in deep formations follows a non-Darcy law. AbuAisha et al.^16^ also conducted a simulation study on H_2_ transport in rock salt and discussed the possible pathways of H_2_ leakage, including viscous flow, diffusion, and dissolution. They concluded that with cyclic H_2_ storage, the amount of H₂ leakage is negligible. Yuan et al.^17^ used He as a proxy for H_2_ to experimentally evaluate the severity of diffusion in rock salt samples from the Lotsberg Formation, in Canada. They concluded that diffusion through pure and intact halite crystals is not significant. However, the presence of impurities such as clays and carbonates in salt samples can create intercrystalline porosity, leading to slightly higher diffusion rates through intergranular open spaces. Strauch et al.^18^ experimentally investigated the diffusivity coefficient of H_2_ in different rocks, including sandstone, clay, and salt. A diffusivity coefficient of 1.3 × 10^− 8^ m^2^/s was obtained for the salt sample, which can be attributed to strong microfractures in the analyzed samples. In a similar study, Song et al.^19^ investigated the diffusion characteristics of salt rock and interlayer mudstone using experiments and numerical studies under various temperature and pressure conditions. They concluded that intergranular cracks and tiny dissolution pores are the main pathways for H_2_ transport, while the interlayer has better petrophysical properties than the salt rock. They reported that the diffusion coefficients of the salt rock cores were between 3.5 × 10^− 10^ and 1.2 × 10^− 9^ m^2^/s.

Given the importance of salt caverns as an ideal site for H_2_ storage and their potential role in advancing clean energy solutions, the mechanism(s) of H_2_ fluid flow within the nanopore structure of salt rocks must be addressed. Furthermore, despite previous efforts, a systematic approach to assess and quantify H_2_ leakage from salt caverns has not been well established. This study seeks to provide accurate formulations and solutions, including effective mechanisms for H_2_ flow through the walls of salt caverns into the surrounding formations. In addition, the study assesses the severity of H_2_ leakage and the mechanisms that contribute to H_2_ transport under different operating conditions. The rest of the paper is organized as follows: First, the theory of H_2_ flow in nanoporous formations is presented, followed by the proposed formulations and the solutions. Existing experimental data are used to validate these formulations, followed by the presentation of the base model with the steps of storage operation. Finally, the H_2_ storage operation within the presented model is investigated, and the impacts of different parameters on the H_2_ leakage rate such as interlayers, maximum operating pressure, and gas type are presented.

## Theory

### Gas flow regime

The Knudsen number (Kn) can be used to categorize different flow regimes of gases in porous rock^20^. It is defined as the ratio of the gas molecular mean free path ($\:\lambda\:$) to the pore diameter (d) and can be written as^21^:

1 $$\:Kn=\frac{\lambda\:}{d},$$

where $\:\lambda\:$ can be calculated using the following formula:

2 $$\:\lambda\:=\:\frac{{K}_{B}T}{\sqrt{2}{\delta\:}^{2}P}.$$

**Fig. 1:**
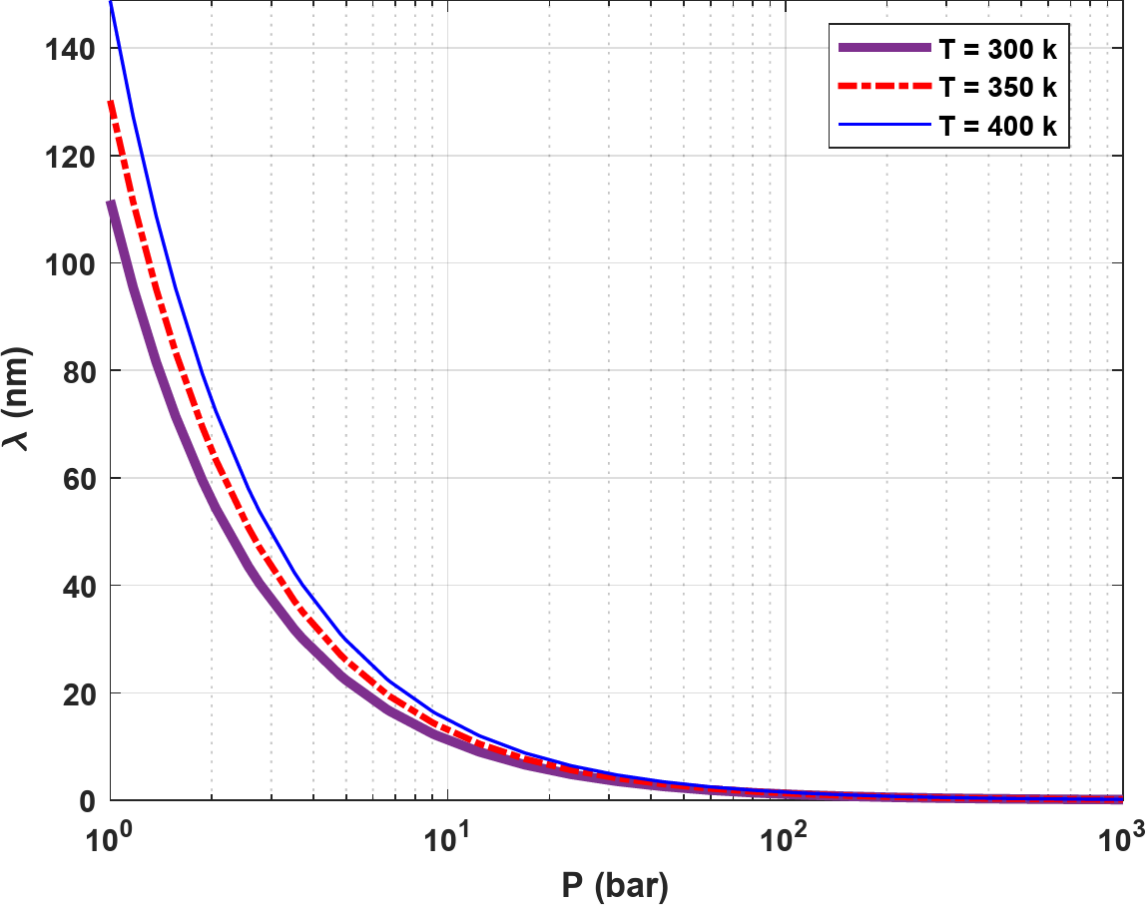
Change of $\:\lambda\:$ of H_2_ with respect to pressure at three temperatures 300, 350, and 400 K.

In the above equation, $\:{K}_{B}$ is the Boltzmann constant (1.3805 × 10^− 23^ J/K), T is the temperature, P is the pressure and δ is the collision diameter of the gas molecule. Using a δ value of 289 pm^22^ for H_2_, $\:\lambda\:$ can be calculated over a pressure range of 1-1000 bar and at three temperatures of 300, 350, and 400 K, as shown in Fig. 1. It can be observed that $\:\lambda\:$ increases with temperature and decreases with pressure. At very high-pressure values, in particular, $\:\lambda\:$ approaches zero.

**Fig. 2:**
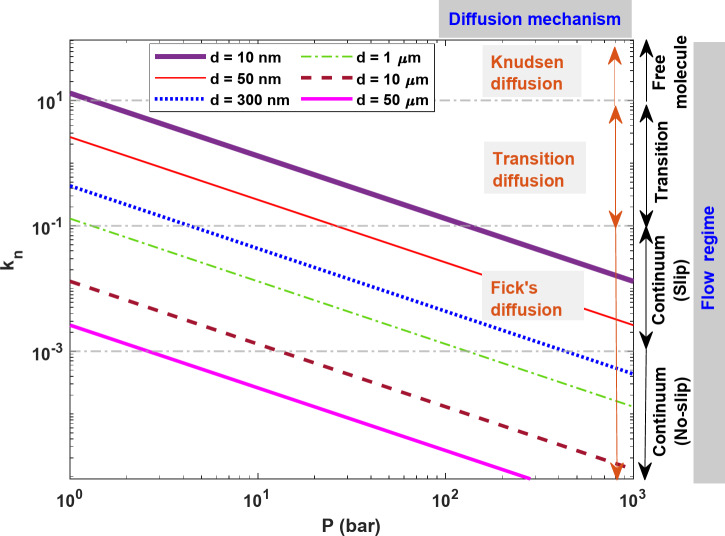
Different flow regimes and mechanisms for H_2_ at various pore diameters and a temperature of 350 K.

Gas flow regimes in the porous media can be categorized into four different regimes based on three important Knudsen numbers: 0.001, 0.1, and 10, which define the boundaries between these regimes^23,24^. If Kn < 0.001, the flow regime is a continuum (no-slip), and the gas flow can be modeled assuming no-slip boundary conditions. Under this condition, Darcy’s law can be applied^20^. At 0.001 < Kn < 0.1, the effect of the gas molecule slippage on the flow can be significant and as such the regime is categorized as continuum (slip). Thus, under this condition, the slip boundary conditions should be considered. At 0.1 < Kn < 10, the gas flow regime is in transition, indicating relatively rarefied gas conditions^25^. Finally, when Kn > 10, the gas flow is a free molecule regime, where collisions between gas molecules can be ignored^26^. Figure 2 shows the plot of Kn versus pressure for various pore diameters (d) and a temperature of 350 K for H_2_.

As it is seen in Fig. 2, at low pressures (less than 2 bar) with 1 μm < d < 50 μm, a continuum (slip) flow regime exists, while the flow regime of H_2_ becomes transitions for d < 1 μm. Conversely, at high pressures exceeding 100 bar, with d > 1 μm, a continuum (no-slip) flow regime exists, and a continuum (slip) flow regime is observed when 1 μm < d < 10 nm.

### Gas diffusion

There are three different mechanisms for molecular diffusion of gases in nanopore formations, including Knudsen diffusion, Fick (bulk) diffusion, and surface diffusion^20^. Surface diffusion refers to the movement of adsorbed gas molecules on the rock surface. Knudsen diffusion, on the other hand, is the consequence of the gas collision with pore walls and when kn > 10 becomes important^26^. The Knudsen diffusion coefficient ($\:{D}_{k}$) can be addressed as^21^:

3 $$\:{D}_{k}=\frac{2r}{3}\sqrt{\frac{8RT}{\pi\:M}},$$

where M represents the molecular weight in kg/mol, R is the molar gas constant (8.314472 J/mol K), and r is the pore radius.

Fick (bulk) diffusion occurs because of collisions between gas molecules and is the prevailing diffusion mechanism when Kn < 0.1. The Fick’s diffusion coefficient ($\:{D}_{F}$) can be defined as^26^:

4 $$\:{D}_{F}=\:\frac{{K}_{B}T}{3\sqrt{2}{\delta\:}^{2}P}\:\sqrt{\frac{8RT}{\pi\:M}}.$$

In conditions where 0.1 < Kn < 10, both Fickian and Knudsen diffusion mechanisms are important, leading to what is known as transition diffusion^20^. Typically, a harmonic averaging approach between $\:{D}_{F}$ and $\:{D}_{K}$ is employed to calculate the transition diffusion coefficient ($\:{D}_{t}$) as below^26^:

5 $$\:{D}_{t}=\:\frac{1}{\frac{1}{{D}_{F}}+\frac{1}{{D}_{k}}}=\:\:\frac{{D}_{F}{D}_{K}}{{D}_{F}{D}_{K}}=\left(\frac{Kn}{Kn+1}\right){D}_{k}.$$

Figure 2 shows also different diffusion mechanisms for H_2_ flow through different pore radii. From this figure, it can be concluded that Knudsen diffusion is only important for H_2_ flow in very tight formations and low-pressure ranges. At a pressure of more than 100 bar and a pore diameter of more than 10 nm, Fick diffusion emerges as the dominant diffusion mechanism.

A new unified gas flow model was proposed by Pang et al.^27^, valid for the entire Knudsen number. The effect of gas slippage, bulk diffusion, Knudsen diffusion, surface diffusion, and cross-sectional geometry of the flow path were also considered in the proposed model. They validated their unified gas flow model in long nanotubes by comparing the results with experimental data from Tison^28^ and the analysis of the Boltzmann equation by Loyalka and Hamoodi^29^. They suggested the following formula for the viscous mass flux ($\:{J}_{visc}$) for circular nano-channels:

6 $$\:{J}_{visc}=\rho\:\frac{{r}^{2}}{8\mu\:}\left(1+\frac{8Kn}{Kn+1}\right)\frac{\varDelta\:P}{L},$$

where ρ is the fluid density, P is the pressure, $\:L$ represents the characteristic length or distance along which the pressure drops ($\:\varDelta\:P$) occurs and $\:\mu\:$ is the fluid viscosity. Moreover, they suggested the following formula to calculate diffusion mass flux ($\:{J}_{D}$):

7 $$\:{J}_{D}=\frac{\rho\:}{P}{D}_{t}\frac{\varDelta\:P}{L}.$$

**Fig. 3:**
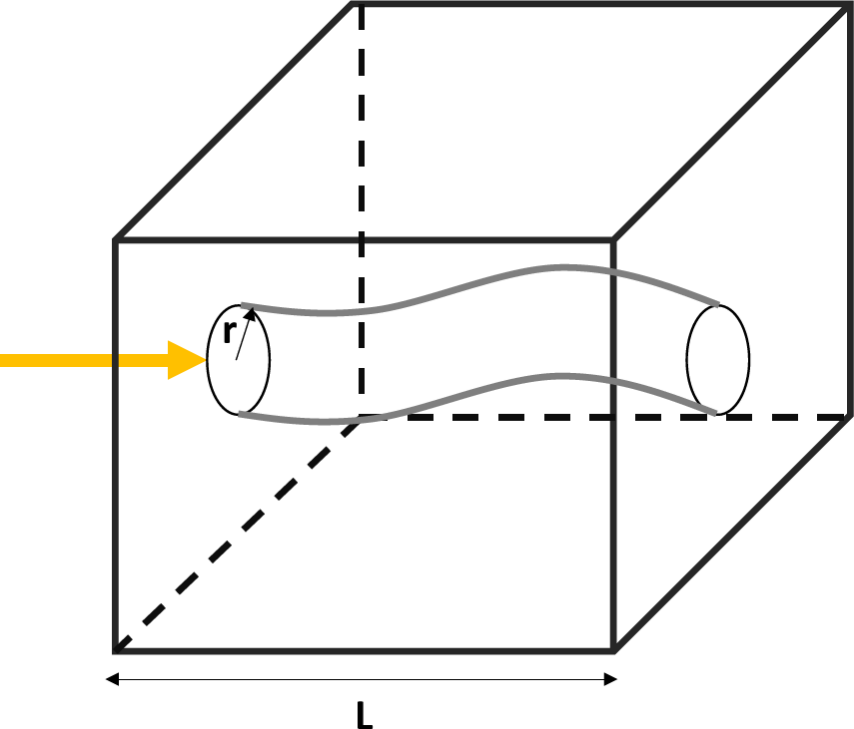
Flow of H_2_ through a tortuous capillary in a salt rock.

In this study, the above unified gas flow model is used to calculate the mass interactions between salt caverns and the surrounding formations. It is important to note that, given the relatively low affinity of H_2_ to the rock surface, surface diffusion was neglected in this study^30^. To incorporate the impact of tortuous flow pathways (see Fig. 3), $\:L$ in Eqs. (6) and (7) should be replaced with the actual flow path ($\:{L}^{*}$) where $\:{L}^{*}$ can be linked to $\:L$ using the definition of tortuosity ($\:{\uptau\:}$) as follows:

8 $$\:{\uptau\:}=\frac{{L}^{*}}{L}.$$

The Bruggeman equation is commonly used to relate $\:{\uptau\:}$ to the formation porosity ($\:\varphi\:$) as follows^31^:

9 $$\:{{\uptau\:}}^{2}=\frac{1}{\sqrt{\varphi\:}}.$$

## Formulations and solution

The governing equation for the gas flow in the x-direction can be written as^32^:

10 $$\:-\frac{\partial\:}{\partial\:x}\left(\dot{{m}_{x}}{A}_{x}\varphi\:\right)\varDelta\:x={V}_{b}\frac{\partial\:\varphi\:\rho\:}{\partial\:t},$$

where $\:\dot{{m}_{x}}$ is the mass flux in the x direction, $\:{A}_{x}$ is the cross-sectional area in the x-direction, and $\:{V}_{b}$ is the bulk volume. Assuming a constant $\:\varphi\:$ with respect to time, the right-hand side of Eq. (10) can be expressed as:

11 $$\:{V}_{b}\frac{\partial\:\varphi\:\rho\:}{\partial\:t}={V}_{b}\varphi\:\frac{\partial\:\rho\:}{\partial\:P}\frac{\partial\:P}{\partial\:t}={V}_{b}\varphi\:\rho\:{C}_{g}\frac{\partial\:P}{\partial\:t},$$

where $\:{C}_{g}$ is the gas compressibility and can be defined as^33^:

12 $$\:{C}_{g}=\frac{1}{\rho\:}\frac{\partial\:\rho\:}{\partial\:P}.$$

Classic gas equation of state can be used to calculate gas density as^34^:

13 $$\:\rho\:=\frac{PM}{ZRT},$$

where T is the absolute temperature in kelvin. The gas unified model introduced before can then be used to calculate the amount of $\:\dot{{m}_{x}}$ as:

14 $$\:\dot{{m}_{x}}=-\frac{1}{\tau\:}\left(\rho\:\frac{{r}^{2}}{8\mu\:}\left(1+\frac{8Kn}{Kn+1}\right){+D}_{t}\frac{M}{ZRT}\right)\frac{\partial\:P}{\partial\:x}.$$

**Fig. 4:**
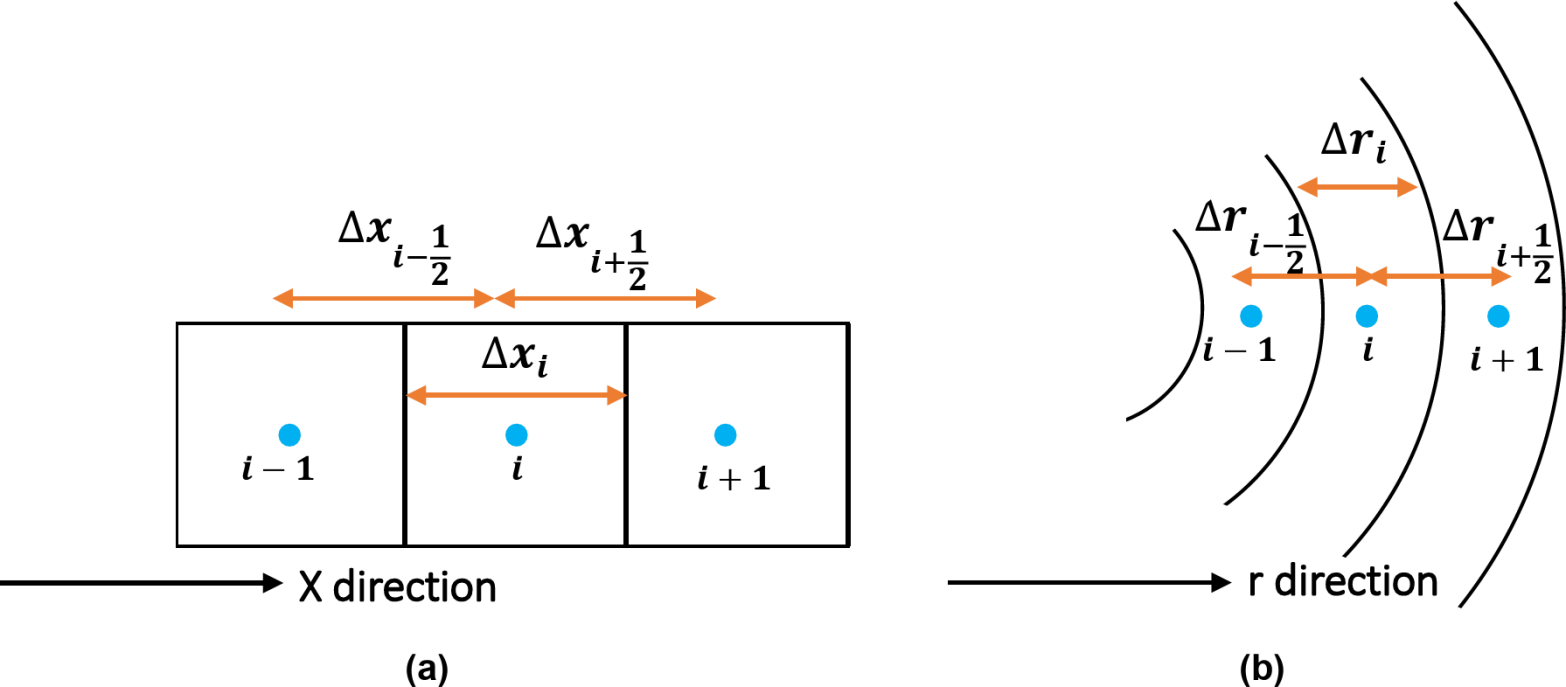
Discretization scheme for one-dimensional block-centered gridding system in (**a**) cartesian and (**b**) radial coordinates.

Figure 4 shows a one-dimensional block-centered discretization scheme in Cartesian and radial coordinates. By using the block-centered gridding and the central finite difference approximation for the spatial derivatives, and backward finite difference approximation for the temporal derivatives, together with an iterative approach to linearize the nonlinearity, the discretization of the governing equation can be expressed as follows:

15 $$\:\left({T}_{i+\frac{1}{2}}^{{n+1}^{\nu\:}}\right){P}_{i+1}^{{n+1}^{\nu\:+1}}-{\left({T}_{i+\frac{1}{2}}^{{n+1}^{\nu\:}}+{T}_{i-\frac{1}{2}}^{{n+1}^{\nu\:}}+{A}_{i}^{{{*}^{n+1}}^{\nu\:}}\right)P}_{i}^{{n+1}^{\nu\:+1}}+\left({T}_{i-\frac{1}{2}}^{{n+1}^{\nu\:}}\right){P}_{i-1}^{{n+1}^{\nu\:+1}}=-{A}^{{{*}^{n+1}}^{\nu\:}}{P}_{i}^{n},$$

where $\:{T}_{i\pm\:\frac{1}{2}}^{{n+1}^{\nu\:}}$ is the transmissibility at the left or right boundaries (see Fig. 4) at time step $\:n+1$ and iteration $\:\nu\:$ and can be presented as:

16 $$\:{T}_{i\pm\:\frac{1}{2}}={\left({\frac{1}{\tau\:\varDelta\:x}A}_{x}\varphi\:\left(\rho\:\frac{{r}^{2}}{8\mu\:}\left(1+\frac{8Kn}{Kn+1}\right){+D}_{t}\frac{M}{ZRT}\right)\right)}_{i\pm\:\frac{1}{2}}.$$

$\:{A}^{*}$ is also defined as:

17 $$\:{A}_{i}^{*}=\frac{{\left({V}_{b}\rho\:{C}_{g}\right)}_{i}}{\varDelta\:t},$$

where $\:\varDelta\:t$ is the time step. As can be seen, $\:{T}_{i\pm\:\frac{1}{2}}^{{n+1}^{\nu\:}}$and $\:{A}_{i}^{{{*}^{n+1}}^{\nu\:}}$are evaluated at the pressures obtained from the previous iteration. The iterations continue until the average difference in error between the pressures at the current and previous iterations falls below a pre-specified threshold. At the start of the iterations, the converged pressures from the previous time step ($\:{P}^{n}$) can be used as the initial guess. For properties such as $\:\rho\:$ and $\:\mu\:$ inside the $\:{T}_{i\pm\:\frac{1}{2}}$, which must be evaluated at boundaries, the average pressure between two adjacent grids can be used.

Furthermore, Eq. (17) in a more compact form can be expressed as:

18 $$\:\left({E}_{i}^{{n+1}^{\nu\:}}\right){P}_{i+1}^{{n+1}^{\nu\:+1}}+{\left({C}_{i}^{{n+1}^{\nu\:}}\right)P}_{i}^{{n+1}^{\nu\:+1}}+\left({W}_{i}^{{n+1}^{\nu\:}}\right){P}_{i-1}^{{n+1}^{\nu\:+1}}={Q}_{i}^{{n+1}^{\nu\:}},$$

where $\:{E}_{i}^{{n+1}^{\nu\:}}$, $\:{W}_{i}^{{n+1}^{\nu\:}}$, $\:{C}_{i}^{{n+1}^{\nu\:}}$, and $\:{Q}_{i}^{{n+1}^{\nu\:}}$can be defined as:

19 $$\:{E}_{i}^{{n+1}^{\nu\:}}={T}_{i+\frac{1}{2}}^{{n+1}^{\nu\:}},{C}_{i}^{{n+1}^{\nu\:}}=-\left({T}_{i+\frac{1}{2}}^{{n+1}^{\nu\:}}+{T}_{i-\frac{1}{2}}^{{n+1}^{\nu\:}}+{A}_{i}^{{{*}^{n+1}}^{\nu\:}}\right),{W}_{i}^{{n+1}^{\nu\:}}={T}_{i-\frac{1}{2}}^{{n+1}^{\nu\:}},{Q}_{i}^{{n+1}^{\nu\:}}=-{A}_{i}^{{{*}^{n+1}}^{\nu\:}}{P}_{i}^{n}.$$

If a pore size distribution is available for a rock sample, the $\:{T}_{i\pm\:\frac{1}{2}}$ can be calculated using the following formula instead of mean pore radius ($\:r$):

20 $$\:{T}_{i\pm\:\frac{1}{2}}=\frac{{\left({\frac{1}{\tau\:\varDelta\:x}A}_{x}\varphi\:{\int\:}_{{r}_{min}}^{{r}_{max}}\left(\rho\:\frac{{r}_{i}^{2}}{8\mu\:}\left(1+\frac{8Kn}{Kn+1}\right){+D}_{t}\frac{M}{ZRT}\right)F\left({r}_{i}\right)d{r}_{i}\right)}_{i\pm\:\frac{1}{2}}}{{\left({\int\:}_{{r}_{min}}^{{r}_{max}}F\left({r}_{i}\right)d{r}_{i}\right)}_{i\pm\:\frac{1}{2}}},$$

where $\:F\left({r}_{i}\right)$ represents the probability of a pore having a radius of $\:{r}_{i}$, while $\:{r}_{min}$ and $\:{r}_{max}$ are the minimum and maximum pore radii, respectively. It should be noted that Eq. (20) take into accounts the influence of pore heterogeneity.

The apparent permeability ($\:k$), the viscous flow component of the apparent permeability ($\:{k}_{V}$), and the diffusion component of the apparent permeability ($\:{k}_{D}$) can be expressed as follows:

21 $$\:k=\frac{\varphi\:}{\tau\:}\frac{{\int\:}_{{r}_{min}}^{{r}_{max}}\left(\frac{{r}_{i}^{2}}{8}\left(1+\frac{8Kn}{Kn+1}\right){+D}_{t}\frac{\mu\:}{P}\right)F\left({r}_{i}\right)d{r}_{i}}{{\int\:}_{{r}_{min}}^{{r}_{max}}F\left({r}_{i}\right)d{r}_{i}},$$

22 $$\:{k}_{V}=\frac{\varphi\:}{\tau\:}\frac{{\int\:}_{{r}_{min}}^{{r}_{max}}\frac{{r}_{i}^{2}}{8}\left(1+\frac{8Kn}{Kn+1}\right)F\left({r}_{i}\right)d{r}_{i}}{{\int\:}_{{r}_{min}}^{{r}_{max}}F\left({r}_{i}\right)d{r}_{i}},$$

23 $$\:{k}_{D}=\frac{\varphi\:}{\tau\:}\frac{{\int\:}_{{r}_{min}}^{{r}_{max}}{D}_{t}\frac{\mu\:}{P}F\left({r}_{i}\right)d{r}_{i}}{{\int\:}_{{r}_{min}}^{{r}_{max}}F\left({r}_{i}\right)d{r}_{i}}.$$

The movement of stored H_2_ from the salt cavern into the surrounding formation can be visualized more realistically with radial coordinates. Figure 4b shows the discretization scheme for one direction (in the r-direction) for a block-centered grid system. The formulation previously proposed for the Cartesian system can be transferred to a radial system by modifying $\:\varDelta\:x$ to $\:\varDelta\:r$ and $\:{A}_{x}$ to $\:{A}_{r}$.

The formulations presented here for modeling H_2_ flow through salt rocks incorporate key mechanisms such as various types of diffusion (Knudsen, transition, and Fickian) and viscous flow, which are crucial for the accurate description of H_2_ transport. In addition, slip flow is considered, as it can play an important role in H_2_ flow across varying pressures and pore sizes (see Fig. 2). The effect of pore heterogeneity is also integrated into the model to better capture the complexity of the storage medium. Furthermore, the numerical simulation scheme explained is widely used and well-established, making its implementation straightforward for future research and applications.

Accurate values for H_2_ transport properties, including viscosity and density, are essential for predicting H_2_ flow through salt rock. In this study, the correlations developed by Wei et al.^34^ was used for H_2_ viscosity and density calculation. These correlations were formulated using symbolic regression, based on 370 experimental data points for density and 1126 for viscosity. The density correlation was also validated against the Leachman equation of state^35^. The proposed viscosity correlation is applicable for temperatures range of 14 to 1000 K and pressures up to 220 MPa. The density correlation offers a high prediction accuracy within the specific ranges of 150–423 K for temperature and 0.1–220 MPa for pressure.

## Validation

**Fig. 5:**
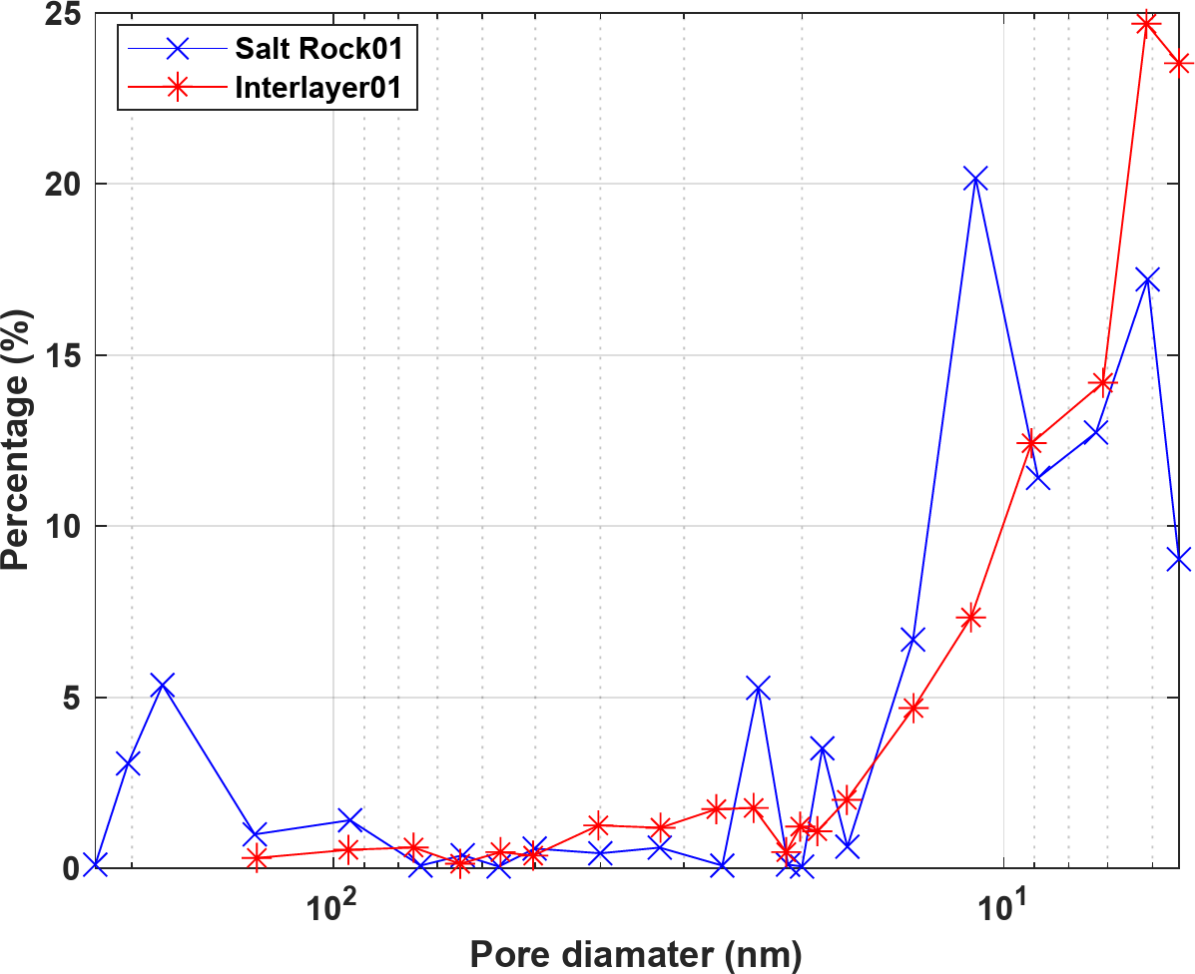
Pore size distributions of samples Salt Rock01 and Interlayer01.

To validate the methodology proposed for simulating H_2_ leakage from formations surrounding salt caverns, the experimental data of Wang et al.^13^ was used. They conducted a series of experiments with core samples of salt rock and interlayers taken from a salt cavern gas storage site in Jintan, China. The experimental setup included upstream and downstream chambers with measurement units recording pressure changes in both chambers. The core samples used had a diameter of 25 mm and a height of 50 mm, while the volumes of the upstream and downstream chambers were 13,376 mL and 14,536 mL respectively. The properties of the two samples Salt Rock01 and mudstone Interlayer01 and the experimental conditions are given in Table 1. Helium (He) was used for Salt Rock01, while nitrogen (N_2_) was used for Interlayer01. Figure 5 shows the pore size distributions of these two samples.

**Table 1:** Properties and experimental conditions of samples Salt Rock01 and Interlayer01.

Sample name	Gas type	Porosity (%)	Tortuosity	Initial upstream pressure (bar)	Initial downstream pressure (bar)
Salt Rock01	He	1.16	3.28	15.1	5.1
Interlayer01	N_2_	5.8	2.71	20.2	10.1

**Fig. 6:**
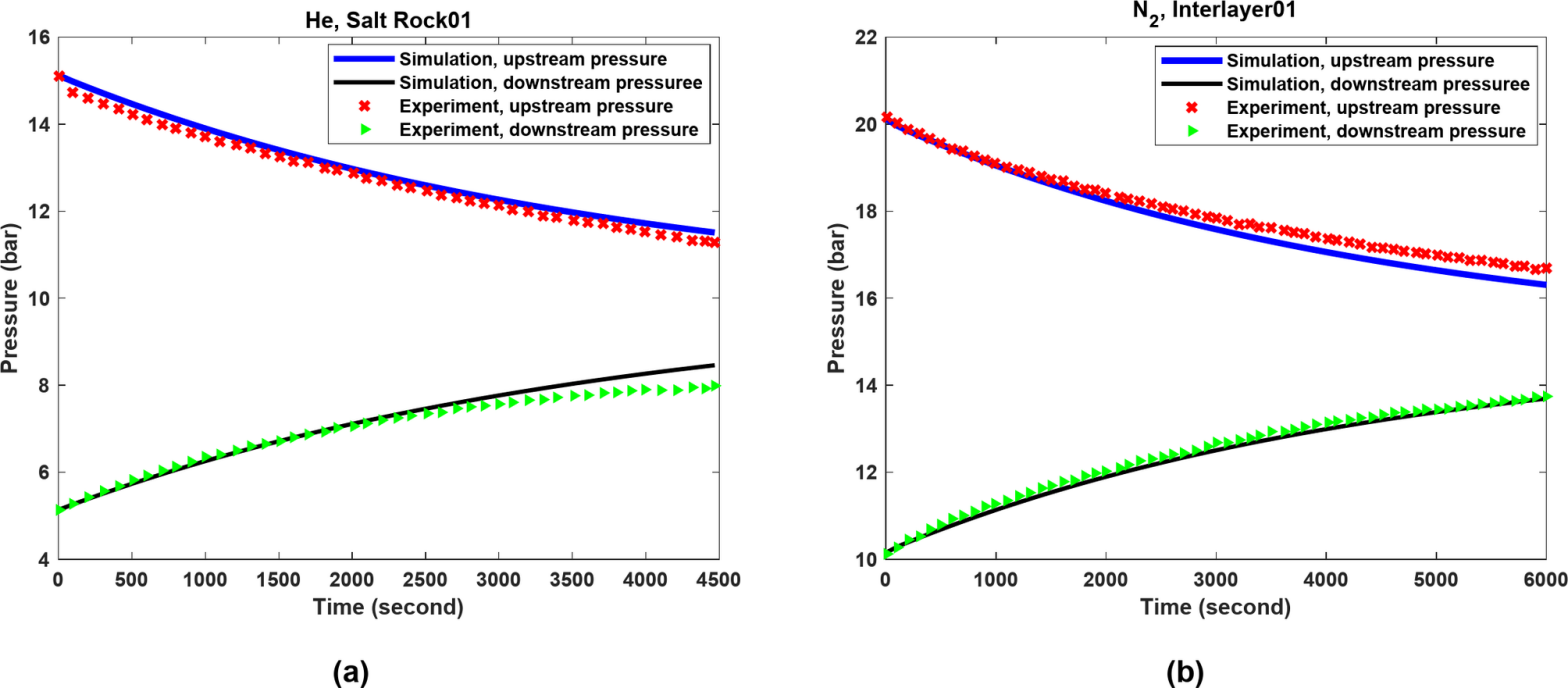
Comparison of the pressure changes in the upstream and downstream chambers for samples (**a**) Salt Rock01 and (**b**) Interlayer01 using experimental and numerical approaches.

To replicate the experimental conditions, a model with 100 grid blocks was used. The Peng-Robinson equation of state was used to calculate the gas densities (N_2_ and He) under the experimental conditions^36^. He and N_2_ were assigned viscosities of 20 µPa s and 19 µPa s, respectively^37^. Figure 6 compares the upstream and downstream pressures of the Salt Rock01 and Interlayer01 samples obtained from the numerical modeling approach which are validated against the experimental data of Wang et al.^13^.

Due to the gas flow (N_2_ and He) from the upstream chamber, there is a pressure drop in the upstream chamber and a pressure increase in the downstream chamber. The pressure predicted by the numerical simulation approach agrees very well with the experimental data. The mean absolute percentage errors for Salt Rock01 and Interlayer01 were 3.49% and 2.18%, respectively.

**Fig. 7:**
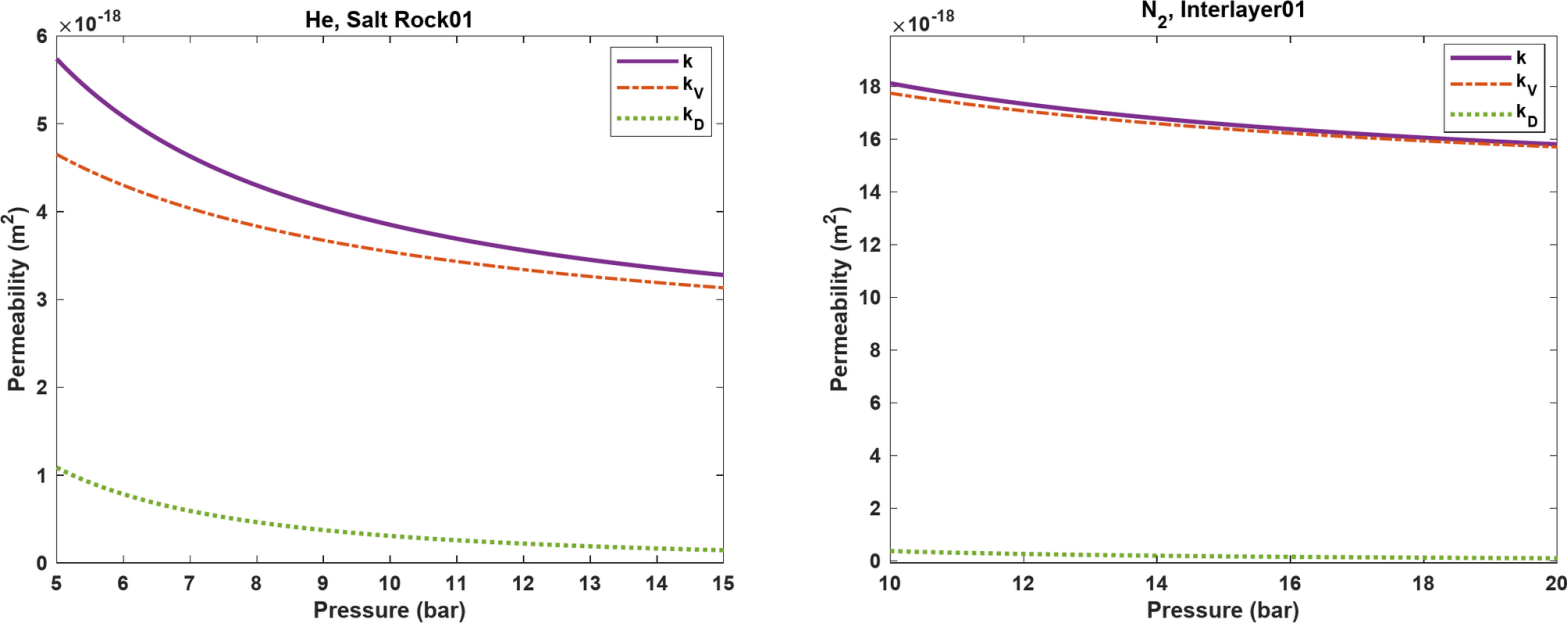
Prediction of $\:k$, $\:{k}_{V}$, and $\:{k}_{D}$ as functions of pressure for (**a**) Salt Rock01 and (**b**) Interlayer01.

Figure 7 shows $\:k$, $\:{k}_{V}$, and $\:{k}_{D}$ for Salt Rock01 and Interlayer01 across the tested pressure ranges (5–15 bar for Salt Rock01 and 10–20 bar for Interlayer01). As can be seen, the contribution of $\:{k}_{V}\:$to $\:k$ is more significant in both cases compared to $\:{k}_{D}$. Particularly, at lower pressures, higher slip flow conditions result in a larger $\:{k}_{V}$, but its impact diminishes as pressure increases. In addition, due to the reduction in $\:\lambda\:\:$with increasing pressure, $\:{k}_{D}\:$also decreases with increasing pressure. At very high pressure, it is expected that $\:k$ is primarily influenced by $\:{k}_{V}$ without the effect of slip flow, as both diffusion and slip flow become negligible under such conditions. It is also worth mentioning that Interlayer01 had relatively better rock properties compared to Salt Rock01, leading to higher $\:k$ values in this type of rock.

## Base model and storage operation

**Fig. 8:**
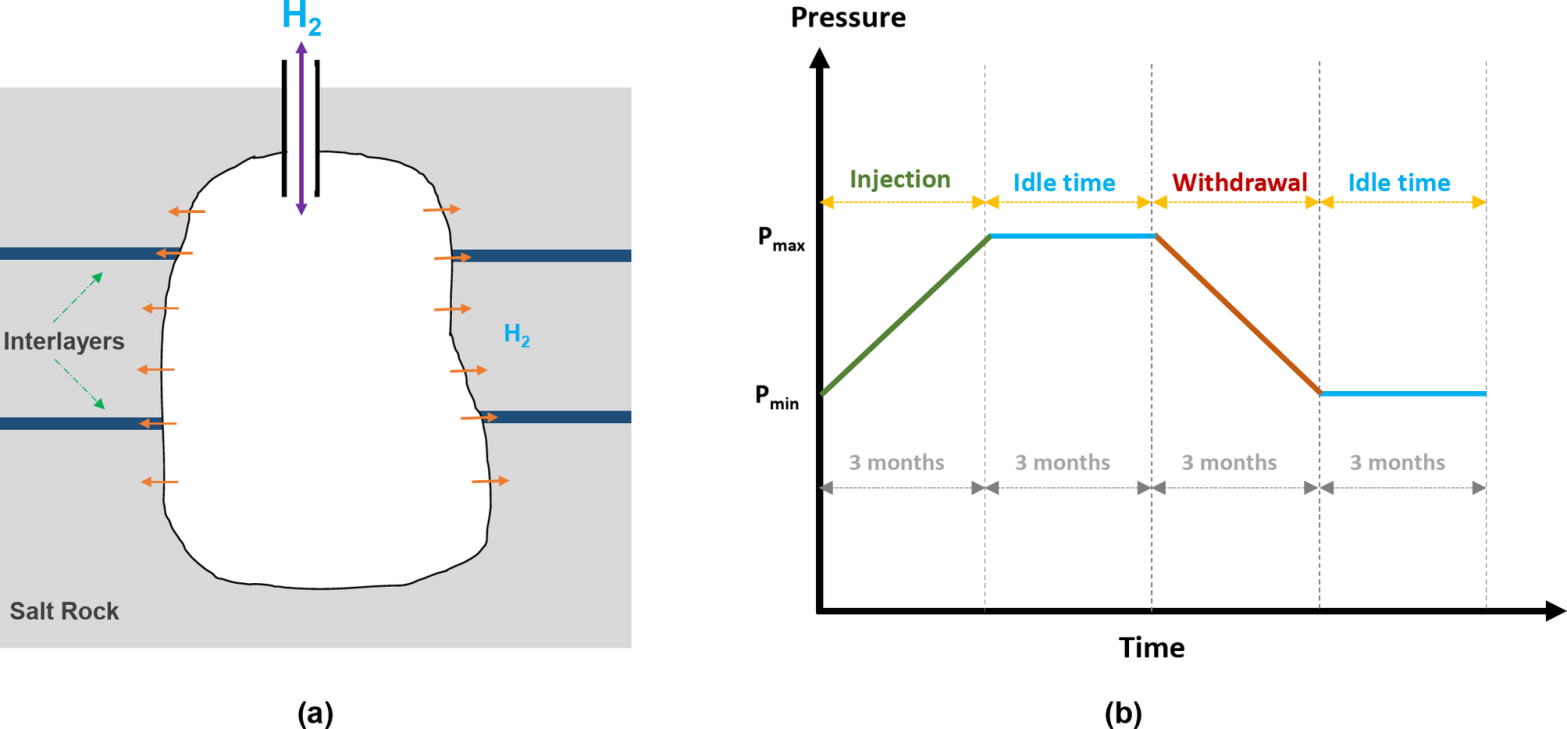
(**a**) Schematic representation of a salt cavern and gas leakage into the surrounding formation, and (**b**) cyclic operation of H_2_ storage in the base model.

Figure 8a shows a schematic representation of the salt cavern and its interactions with the surrounding geological formation. To investigate H_2_ leakage, a base model was considered, with its properties detailed in Table 2. The cavern had a height of 120 m and a diameter of 60 m. It was assumed that there was a sump of 12 m at the bottom of the salt cavern. A standard temperature gradient of 0.03 °C/m was used to calculate the temperature of the surrounding formation, assuming a surface temperature of 15 °C, resulting in a temperature of 45 °C. In addition, a pressure gradient of 0.118 bar/m was assumed to calculate the pore pressure of the surrounding formation, resulting in an initial pore pressure of 119 bar. This pressure gradient, which corresponds to a brine density of 1200 kg/m^3^, was used in previous studies to calculate the surrounding pore pressure of salt caverns^16,38^. Considering the volume occupied by the sump, the base model offers a volume of 3.054 × 10^5^ m^3^ with an H_2_ density of 9.68 kg/m^3^ at the maximum salt cavern pressure, making the total capacity of the salt cavern to be 2.955 × 10^6^ kg H_2_. The cyclic H_2_ storage operation in the base model is shown in Fig. 8b. A cyclical storage period of 30 years was considered, in which the pressure in the salt cavern fluctuates between maximum (135 bar) and minimum pressure (70 bar). The injection and production intervals each have a length of 3 months, followed by 3 months of idle time. It should be noted that the depth and operating pressures correspond to those of the Clemens salt cavern (USA), which is used for pure H_2_ storage^9^.

**Table 2:** Base case properties.

Parameter	Value
Salt cavern diameter (m)	60
Salt cavern height (m)	120
Temperature (°C)	45
Sump height (m)	12
Maximum salt cavern pressure (bar)	135
Minimum salt cavern pressure (bar)	70
Surrounding pressure (bar)	119
Porosity (%)	1
Mean pore radius (nm)	15
Depth (m)	1000
Tortuosity	3.16
Simulation time (year)	30

## H_2_ storage in the base model

**Fig. 9:**
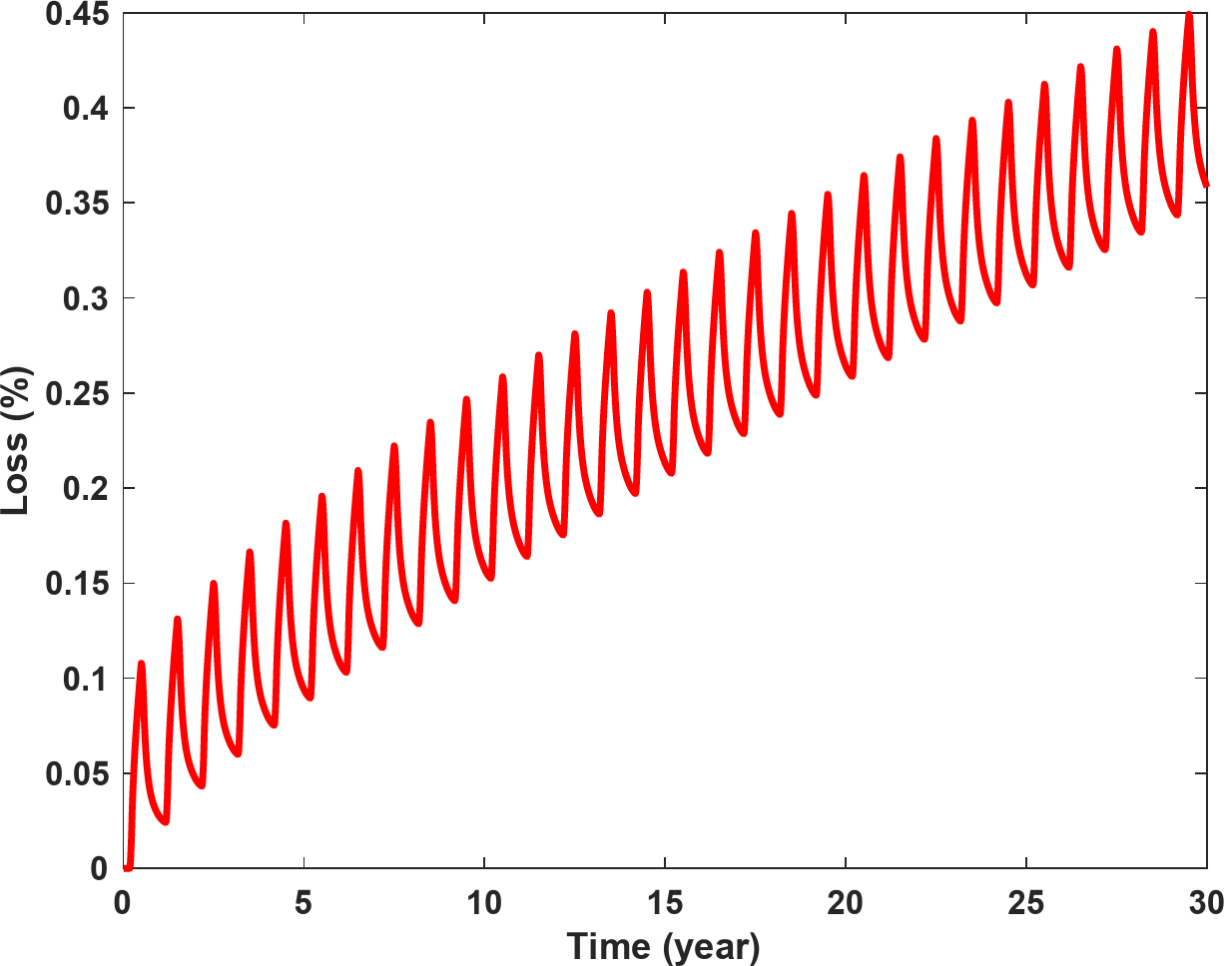
H_2_ loss over 30 years of cyclic storage in the base model.

The amount of H_2_ loss over 30 years of cyclic storage in the base model is shown in Fig. 9. The percentage loss in this figure and the following analysis is the ratio of the cumulative mass of H_2_ leaked into the surrounding formation divided by the maximum capacity of the salt cavern. In the base model, for example, the maximum capacity was 2.955 × 10^6^ kg H_2_. After 30 years of storage, the loss was 0.36%, which corresponds to 10.638 tonnes of H_2_.

**Fig. 10:**
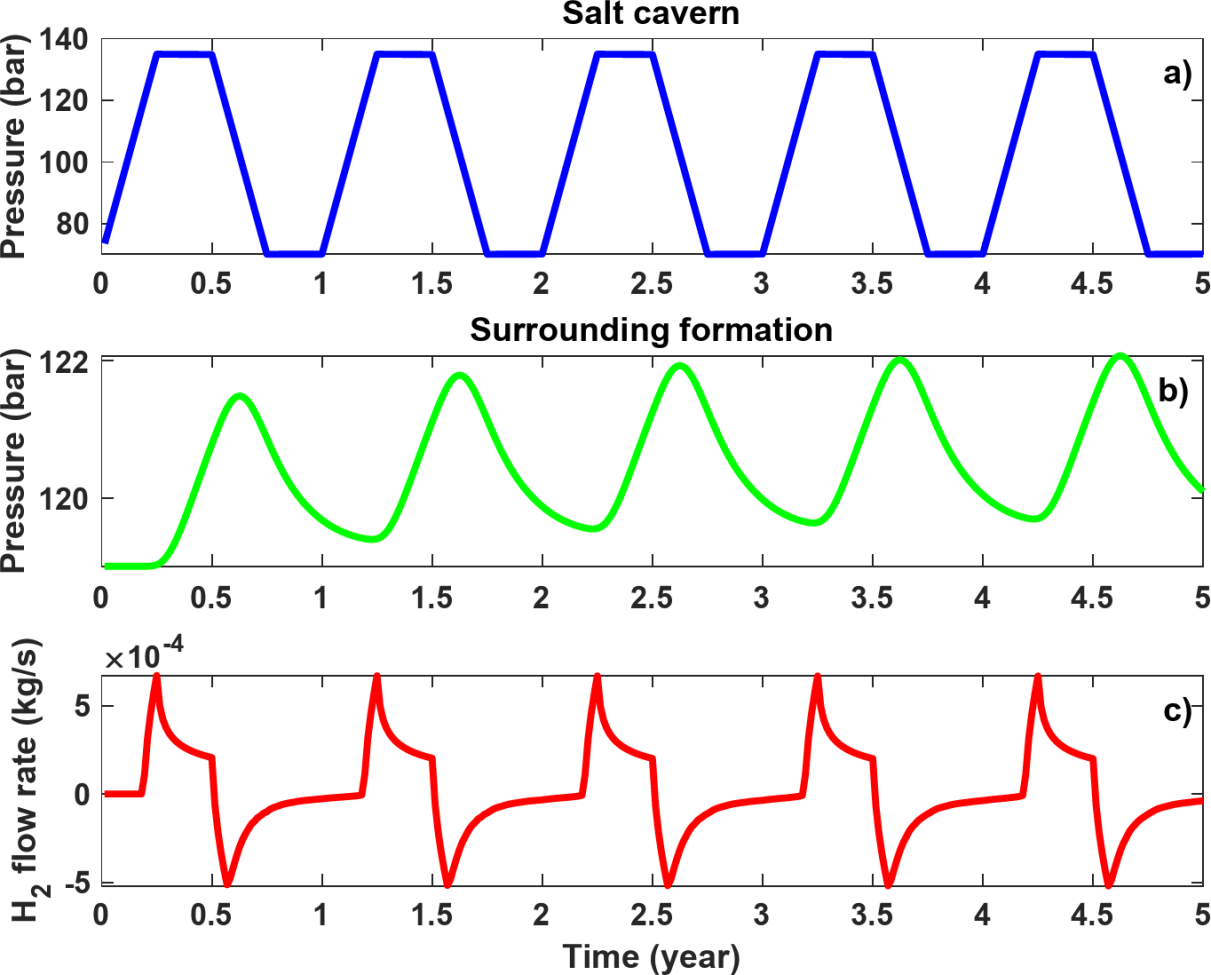
H_2_ storage in the base model over 5 years of cyclic storage: (**a**) pressure in the salt cavern, (**b**) pressure in the surrounding formation 25 m away from the salt cavern wall, and (**c**) H_2_ flow rate between the salt cavern and formation (positive values indicate flow of H_2_ from the salt cavern into the formation, and negative values indicate the flowback).

The fluctuations in the H_2_ loss profile, shown in Fig. 9, are induced by the backflow of leaked H_2_ from the surrounding formation into the salt cavern when the pressure in the salt cavern falls below the formation pressure, e.g. during production followed by idle times. The pressure changes within the salt cavern and at a point 25 m from the salt cavern wall along with the mass rate of H_2_ between the salt cavern and the surrounding formation during the first five cycles are shown in Fig. 10.

It should be noted that a positive mass flow rate in Fig. 10c indicates a flow from the salt cavern into the surrounding formation, while negative values indicate the opposite. The pressure in the salt cavern (Fig. 10a) follows the previously explained pattern designed for cyclic storage of H_2_ in the base model. The pressure in the surrounding formation and 25 m from the salt cavern wall (Fig. 10b) increased from its initial value of 119 bar. It took some time for the leaked H_2_ to reach this point, causing the pressure to rise during injection and the subsequent idle phase. However, due to the backflow of H_2_ during the production periods, there was a drop in pressure and these fluctuations can be observed in all five cycles. A general slight increase in pressure can be observed, which is due to a gradual increase in the cumulative amount of leaked H_2_ in the surrounding formation over time. The maximum H_2_ flow rate into the surrounding formation in the first cycle was 6.72 × 10^− 4^ kg/s, equivalent to 58.06 kg/day. Conversely, the maximum backflow rate was − 5.15 × 10^− 4^ kg/s, which corresponds to − 44.50 kg/day. It can be concluded that, fortunately, most of the leaked H_2_ (due to viscous and diffusion mechanisms) flows back into the salt cavern due to the pressure fluctuations in the salt cavern during cyclic storage operation. If the integrity of the salt allows the cavern to be operated at a lower operating pressure, the backflow rate will increase. As a result, the H_2_ loss could be even lower. It should be emphasized that the reported loss is not based on the cumulative amount of H_2_ injected over 30 years, but on the maximum capacity of the salt cavern. If the loss were calculated based on the cumulative amount of H_2_ injected, the calculated loss would be much lower, equal to 0.025%.

**Fig. 11:**
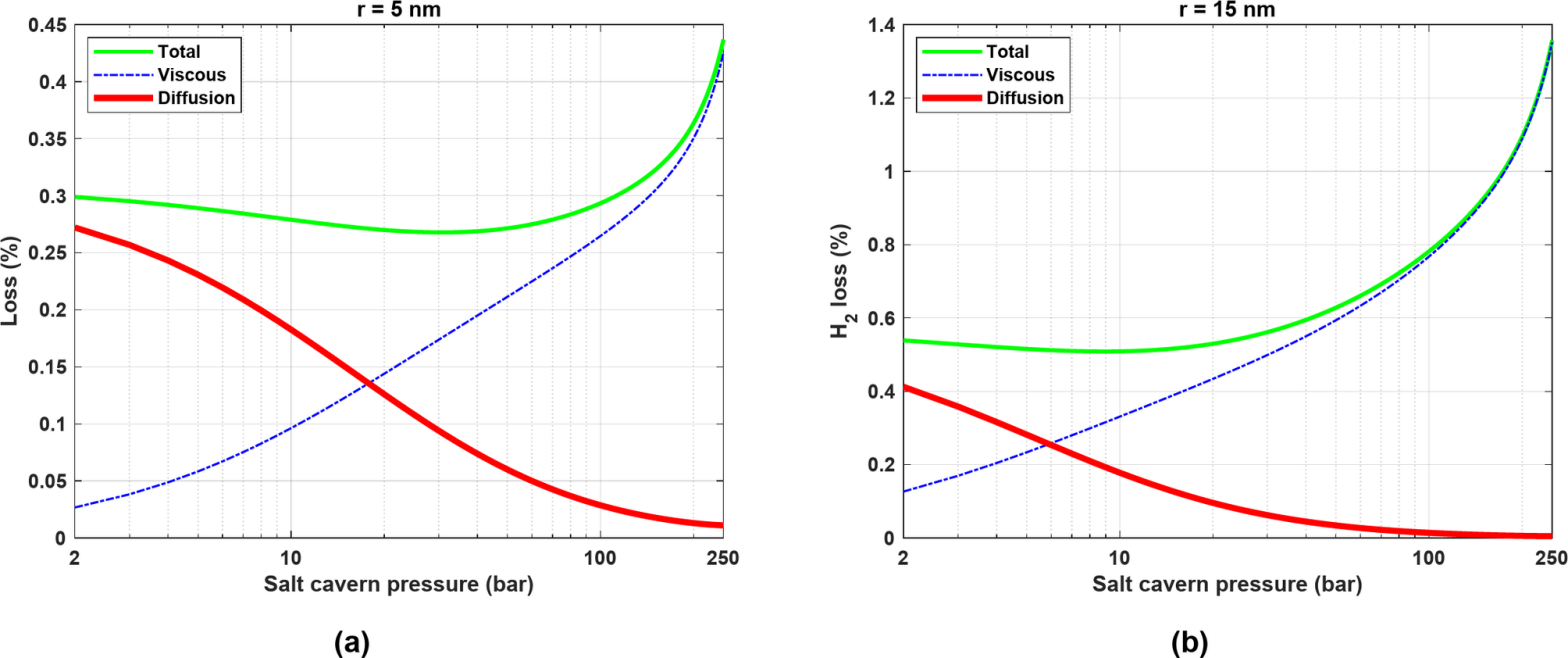
Impact of pressure on the relative contributions of viscous and diffusion mechanisms on H_2_ leakage: (**a**) *r* = 5 nm and (**b**) *r* = 15 nm.

In the base model, the contribution of the diffusion mechanism to H_2_ leakage was negligible, suggesting that viscous flow is primarily responsible for H_2_ loss. To further evaluate the relative effects of the viscous and diffusion mechanisms on H_2_ leakage, the pressure of the salt cavern in the base model was varied between 2 and 250 bar, with the pressure of the surrounding formation assumed to be half the pressure of the salt cavern. Assuming no cyclic operation and considering two pore radii of 5 nm and 15 nm, the relative contributions of these two transport mechanisms were compared after 1 year, as shown in Fig. 11.

As can be seen in Fig. 11, for both pore radii, especially for the 5 nm case, diffusion is the dominant mechanism of H_2_ leakage at very low pressure. With increasing pressure, the influence of viscous flow increases, and at very high pressure, viscous flow becomes the predominant means of H_2_ transport into the surrounding formation. For example, at a pore radius of 15 nm, the contribution of diffusion is negligible when the pressure exceeds 100 bar. However, at a pore radius of 5 nm, diffusion still has a small influence on H_2_ loss even at very high pressures. These observations are consistent with the results reported by Wang et al.^13^.

As the pressure increases, $\:\lambda\:$ decreases and approaches zero, as expressed by Eq. (2) and shown in Fig. 1. As a result, Kn also decreases and approaches zero at very high pressures, which in turn decreases $\:{D}_{t}$. Finally, the flux due to diffusion becomes negligible at very high pressures. This effect is more significant in formations with larger pore radii, as the value of Kn is even lower in these formations (Kn is inversely proportional to the pore radius). It should be emphasized that $\:{J}_{visc}$ is directly proportional to the square of the pore radius. As a result, the losses due to viscous flow are more pronounced with a larger pore radius of 15 nm.

### Interlayers

Salt domes and bedded salt deposits are formed through the evaporation of ancient seas, leaving behind layers of salt and other evaporite minerals (see Fig. 8a). Over time, these layers can become interbedded with sediments such as mudstone and anhydrite due to changes in the depositional environment. As a result, salt caverns are not only composed of halite, but other impurities (interlayers) such as clay/claystone, carbonates, and anhydrite are also observed in the salt cavern structures^13,39,40^. These interlayers may have different rock properties compared to the nanopore-structured salt rock, particularly in terms of porosity and pore radius. Therefore, their impact on the amount of H_2_ loss needs to be carefully evaluated.

**Fig. 12:**
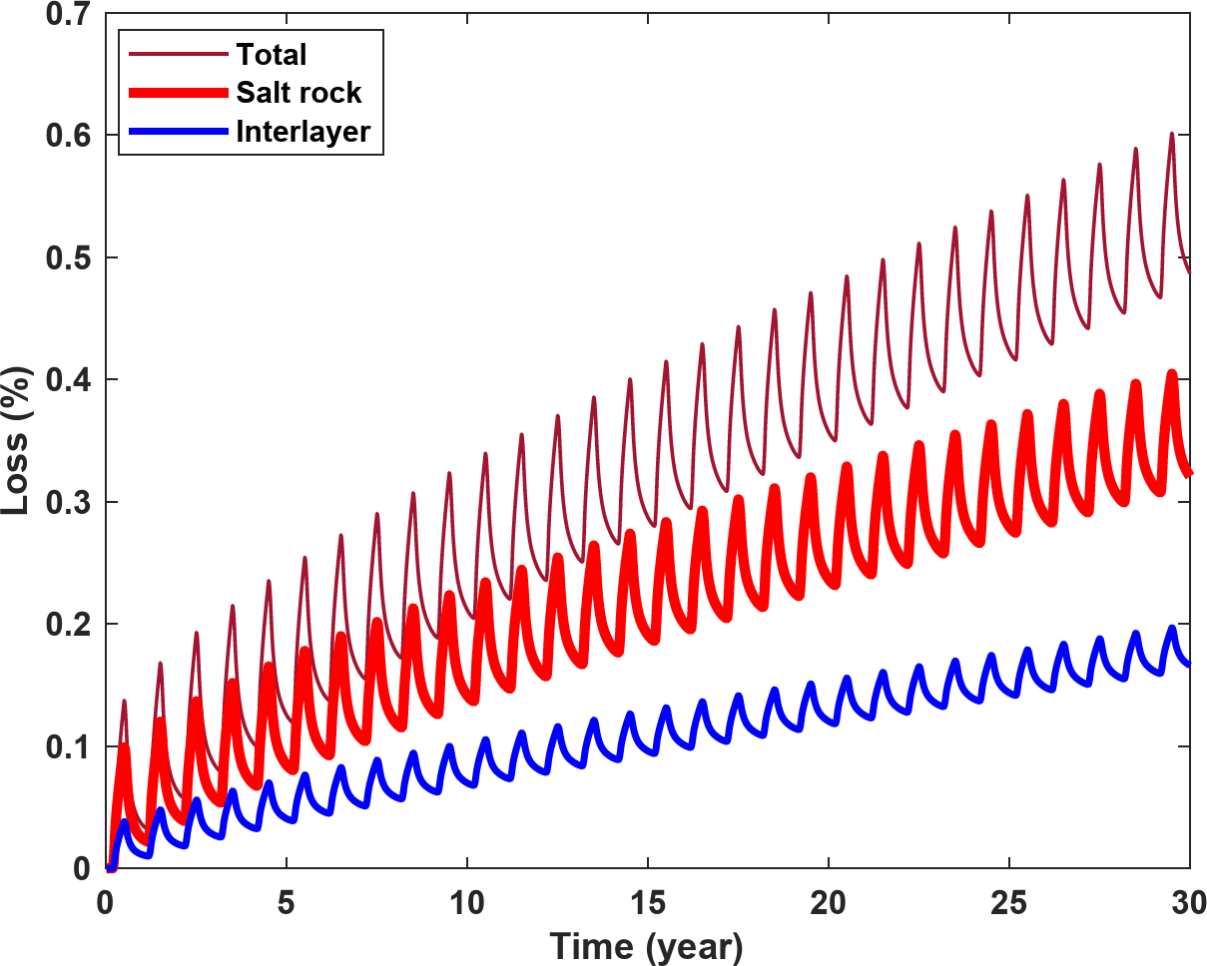
H_2_ loss in the salt rock and interlayer over 30 years of cyclic storage.

To investigate the impacts of these interlayers, an interlayer with a thickness of 3 m, a pore radius of 30 nm, and a porosity of 5% was considered. These values are close to the reported values in previous studies^39,41^. The interactions between the salt rock and the interlayer were neglected due to the one-dimensional simulation approach. However, both the salt rock and the intermediate layer were able to communicate with each other via the salt cavern. Figure 12 shows the amount of H_2_ losses in the salt rock and in the intermediate layer over 30 years of cyclic storage. The final losses after 30 years were 0.17% for the interlayer and 0.32% for the salt rock. The total loss was therefore 0.49%, which corresponds to 14.47 tonnes of H_2_. It is important to note that even a 3-meter interlayer with higher porosity and pore radius could significantly increase the loss, resulting in a 36% increase in total loss (from 0.36 to 0.49%). As described earlier, fluctuations in the loss trend can be linked to the flowback of H_2_ at times when the pressure in the salt cavern is lower than the formation pressure.

**Fig. 13:**
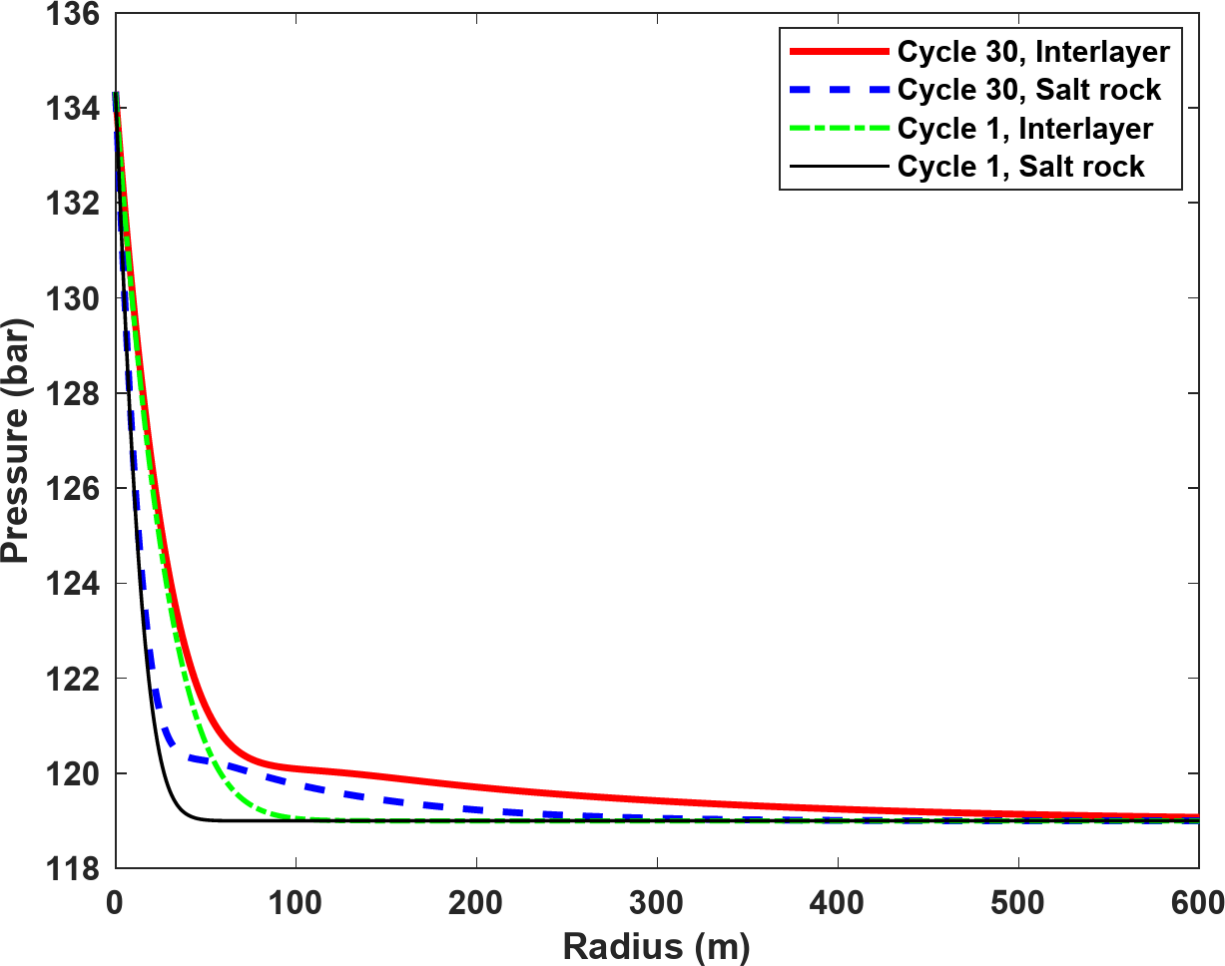
Leakage range within the salt rock and interlayer during the first and 30th cycles.

In addition to the leakage rate and the amount of loss, the leakage range is a decisive factor in the determination of suitable locations for multiple salt caverns within a geological setting. Figure 13 shows the leakage in the salt rock and the interlayer during the 1st and 30th cycles. As it is seen, the leakage in both the salt rock and the interlayer increases over successive cycles. In particular, the leakage range in the interlayer was significantly greater than in the salt rock. During the 30th cycle, for example, the leakage range in the salt rock was about 200 m, while in the interlayer it exceeded 400 m, measured from the wall of the salt cavern. Therefore, the impacts of these interlayers must be carefully analyzed when locating multiple salt caverns within the same salt layer. It is also important to note that most of the leaked H_2_ remained concentrated around the salt caverns, i.e. within 100 m of the cavern wall (see Fig. 13). Therefore, the reported leakage ranges indicate the distance that even a small amount of H_2_ could travel.

To further evaluate the impacts of interlayer types on the extent of H₂ loss, three different interlayers were selected based on a previous study^42^: gray anhydrite (7% porosity and mean pore radius of 79 nm), gypsum mudstone (3% porosity and mean pore radius of 42 nm), and limestone mudstone (1% porosity and mean pore radius of 25 nm). It is worth noting that the thickness of the interlayers was kept constant at 3 m, and the equation proposed by Bernabé^43^ was used to relate porosity, permeability, and mean pore radius in these interlayers.

**Fig. 14:**
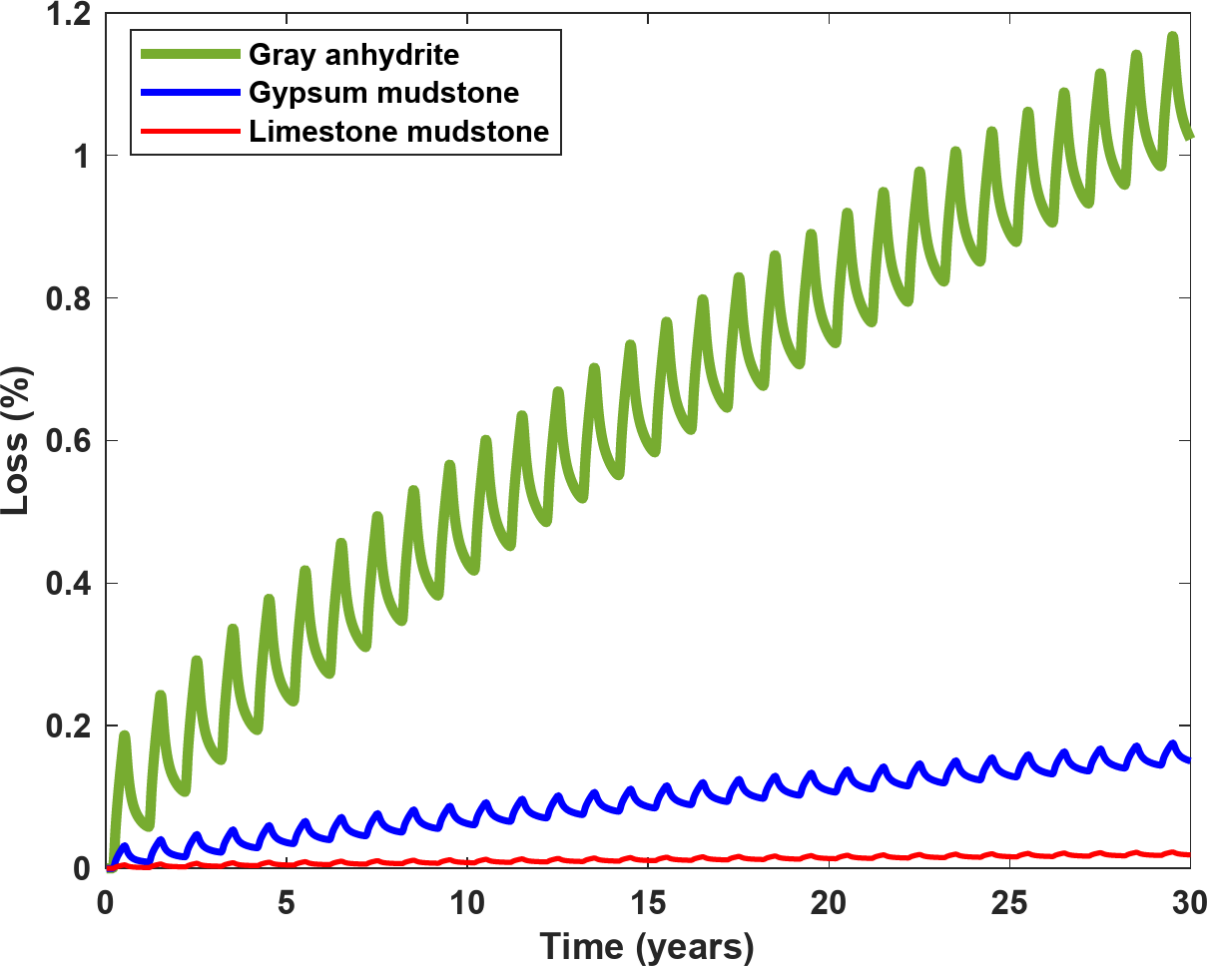
Impact of interlayer types, including gray anhydrite, gypsum mudstone, and limestone mudstone, on the extent of H_2_ loss.

Figure 14 illustrates the effects of interlayer types on H_2_ loss during 30 years of cyclic storage. As shown, the final H_2_ losses for gray anhydrite, gypsum mudstone, and limestone mudstone were 1.02%, 0.15%, and 0.02%, respectively. While the loss for gray anhydrite was considerable, the loss for limestone mudstone was negligible. These results emphasize the significant influence of pore radius and porosity in the interlayer on H_2_ loss. The viscous force, the primary leakage mechanism at high operating pressure, is directly proportional to the square of the mean pore radius of the salt rock (Eq. 6). Consequently, interlayers with larger mean pore radii led to higher H_2_ losses in the base model. In addition, higher porosity provides more space for H_2_ flux from the salt cavern walls, where interlayers are present, and increases the number of capillary tubes contributing to H_2_ transport. As a result, greater H_2_ leakage was observed in interlayers with higher porosity. These results underscore the importance of accurate characterization of the surrounding formation to better estimate H_2_ loss.

### Operating pressure

**Fig. 15:**
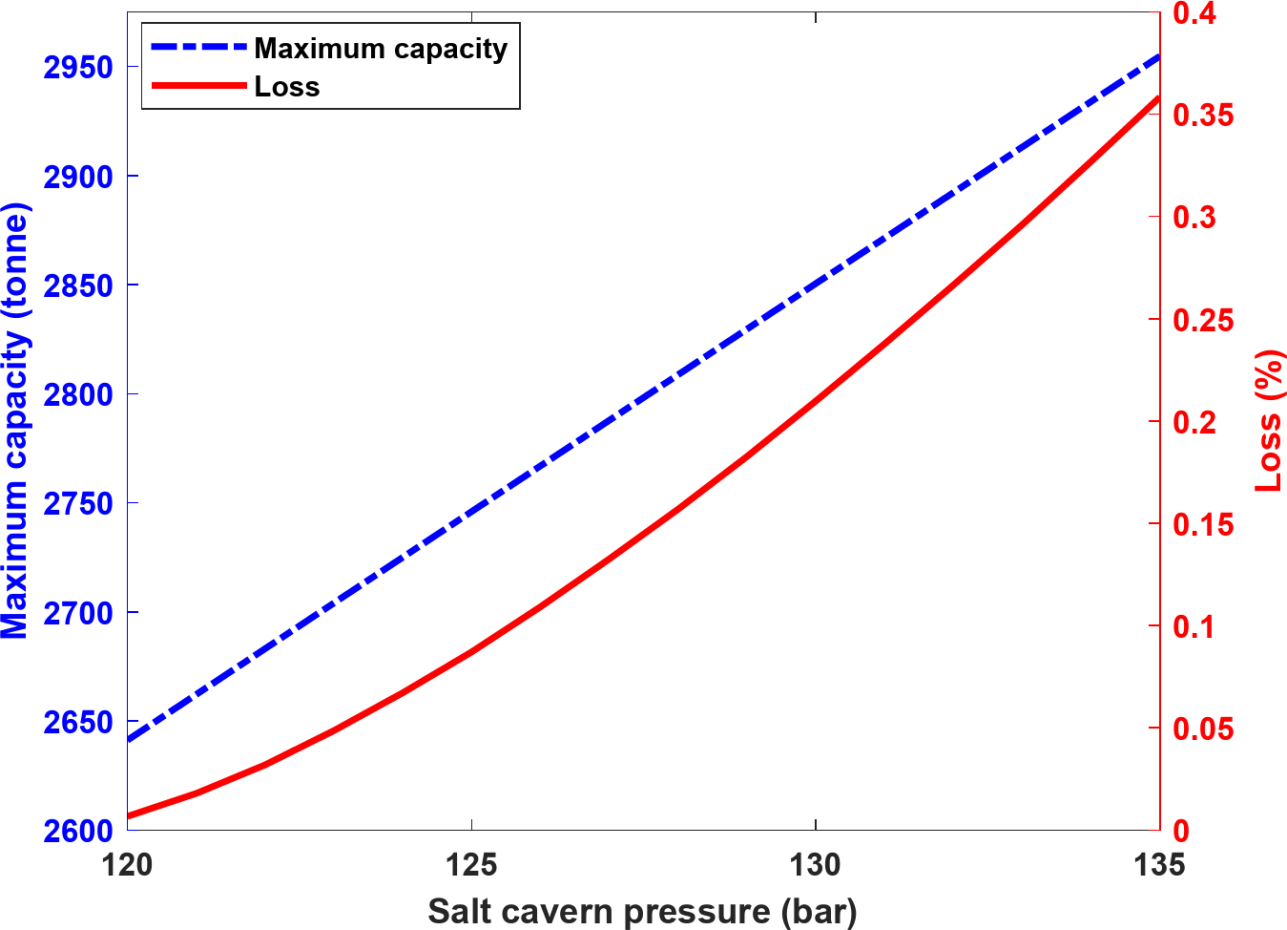
Impact of maximum operating pressure on the maximum capacity of the base model and H_2_ loss after 30 years of cyclic storage.

One of the most important design parameters of salt caverns is their operating pressure, which depends on various factors such as safety and stability. The maximum allowable operating pressure is usually defined based on the fracture initiation pressure^44^. Due to the higher H_2_ density at higher pressure, choosing a very high operating pressure can increase the storage capacity of the salt cavern. However, this may also affect the amount of leakage caused by the pressure difference between the formation pressure and the salt cavern, which in turn affects H_2_ leakage rates. In this section, the effect of maximum operating pressure on the maximum capacity of the base case was analyzed to evaluate the amount of H_2_ loss after 30 years of cyclic storage, as shown in Fig. 15. As can be seen in this figure, the maximum operating capacity of the salt cavern increased from 2,641 × 10^3^ tonnes to 2,955 × 10^3^ tonnes when the maximum operating pressure was increased from 120 to 135 bar. This corresponds to an increase in maximum storage capacity of 11.9%. However, the H_2_ loss at 120 bar was 0.007%, and this value increased to 0.36% at 135 bars, indicating a significant increase in the amount of loss. This could be attributed to the fact that the change in H_2_ density due to the increase in pressure is not significant. In fact, in the base model, the H_2_ density at 120 and 135 bar was 8.65 and 9.68 kg/m^3^ respectively. Therefore, the change in the maximum storage capacity due to the pressure increase was limited. However, the higher-pressure difference between the salt cavern and the surrounding formations increases viscous forces (and the diffusion to some extent), resulting in greater H_2_ leakage. As a result, the effect of the operating pressure on the amount of H_2_ loss should be considered when planning a salt cavern operation.

### Natural gas storage comparison

**Fig. 16:**
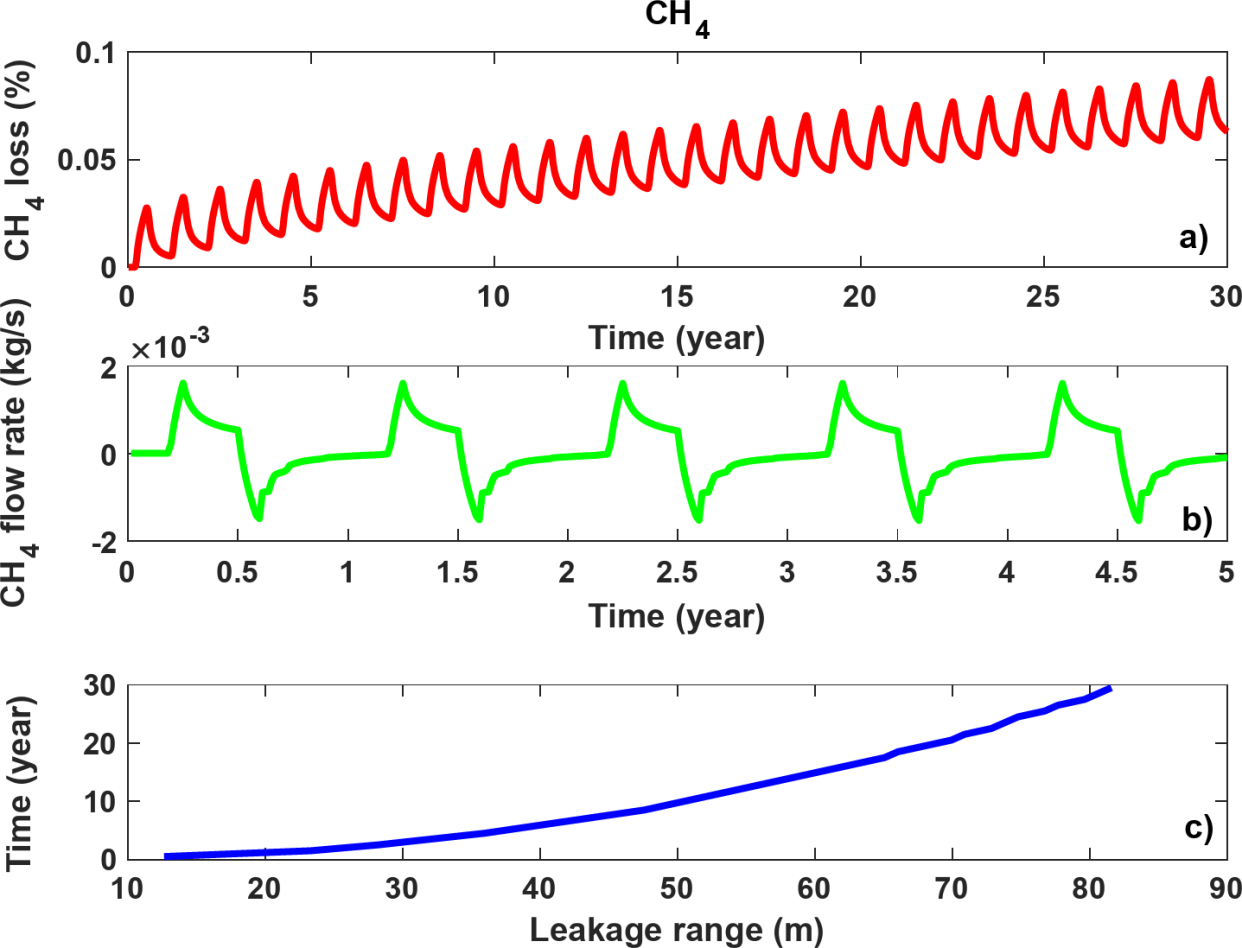
CH_4_ storage in the base model over 30 years of cyclic storage: (**a**) Percentage loss, (**b**) CH_4_ flow rate between the salt cavern and the formation (positive values indicate flow from the salt cavern into the formation, while negative values indicate the backflow), and (**c**) leakage range.

To compare the impacts of the different gases on leakage and storage performance, attempts were made to assess CH_4_ storage using the base model. The only difference to the H_2_ storage model was the type of gas stored, which in this case was CH_4_. To predict the density of CH_4_, the Peng-Robinson equation of state was used^36^. In addition, an average viscosity of 13 µPa s was assumed for CH_4_ based on the operating pressure and temperature of the storage process^45^. It should be noted that the average viscosity of H_2_ in the model was about 9 µPa s. Thus, CH_4_ was more viscous than H_2_ under the operating conditions. The maximum capacity of the salt cavern for the storage of CH_4_ was 2.942 × 10^4^ tonnes, which is about ten times higher than the capacity for H_2_, as the density of CH_4_ at maximum operating pressure is 96.36 kg/m^3^ (compared to the density of H_2_ under the same conditions, which is 9.68 kg/m³). Figure 16 shows the loss of CH_4_ together with the flow rate between the salt cavern and the surrounding formation, as well as the leakage range of CH_4_ during 30 years of cyclic storage. As described earlier, positive mass rates indicate flow from the salt cavern into the formation and vice versa.

The CH_4_ loss after 30 years (Fig. 16a) was 0.063%, which is significantly less than the 0.36% loss observed with H_2_ storage. It should be noted that the mass of CH_4_ leaked (18.556 tonnes) was greater than that of H_2_ (10.638 tonnes), but as the maximum salt cavern capacity for CH_4_ was much higher than that for H_2_, the percentage loss for CH_4_ was much lower.

Figure 16b shows the flow rate between the salt cavern and the surrounding formations. The backflow rate of leaked CH4 is responsible for the fluctuations in CH_4_ loss shown in Fig. 16a. Considering the higher density of CH_4_ compared to H_2_ and the fact that viscous flow is directly proportional to fluid density (Eq. 6), CH_4_ flow rates were higher than those of H_2_. Furthermore, although more CH_4_ leaked into the surrounding formations compared to H_2_ (see Fig. 16c), the higher viscosity of CH_4_ resulted in a leakage range of about 80 m after 30 years, which was much less than the that observed for H_2_. This suggests that the leakage range for H_2_ may be a more critical factor to consider when designing salt caverns.

## Conclusions

In this study, the extent of hydrogen leakage from the walls of salt caverns into the surrounding formations was assessed. A formulation was presented based on a recently introduced unified gas flow model that accounts for gas slippage, bulk diffusion, and Knudsen diffusion and is valid for the entire range of Knudsen numbers. Existing experimental data were used to validate this formulation. The results obtained showed that for a wide range of pore radii and the common temperature and pressure conditions of underground storage sites, slippage flow must be considered for hydrogen flow in salt rocks, as the Knudsen number ranges from 0.001 to 0.1. In a salt cavern with the corresponding dimensions and operating conditions, only 0.36% of the maximum storage mass capacity has leaked into the surrounding formation after 30 years of cyclic storage. Most of the leaked hydrogen would flow back in times when the pressure in the salt cavern falls below the pressure in the formation.

At high operating pressures, the mean free path of the gas molecules is reduced, restricting diffusion flow, and making viscous flow the dominant mechanism due to the higher density of hydrogen at high pressures. Even very thin interlayers with improved rock properties compared to salt rock can significantly influence the amount of leakage, with the leakage range in the interlayer being much larger than in the salt rock. The viscous flow is directly proportional to the square of the pore radius, resulting in more leakage at larger pore radii. In addition, a higher porosity led to greater leakage, as higher porosity values provide more space on the salt cavern wall for hydrogen flow. Increasing the maximum operating pressure of the salt cavern led to a slight increase in the maximum storage mass capacity, as the density of the hydrogen does not increase significantly with pressure. However, the amount of leakage increased significantly. While the storage of natural gas resulted in more leakages compared to hydrogen, the maximum capacity of the salt cavern was almost ten times higher for natural gas than for hydrogen. Consequently, the leakage percentage relative to the maximum capacity was much lower for natural gas storage. Furthermore, the leakage range for natural gas storage was lower compared to hydrogen due to the higher viscosity.

The formulations and numerical simulation scheme presented in this work can estimate hydrogen leakage in salt caverns and evaluate the key parameters that influence leakage rates. The prediction of leakage ranges can also determine the suitable placement of multiple caverns and the width of pillars within a salt structure. Future studies could explore interactions between salt rock and interlayers, multiphase flow within the salt rock, and contacts with the sump as potential areas for further investigation.

## Data Availability

The authors declare that the data supporting the findings of this study are available within the paper.

## References

[1] Ghaedi, M., Østebø Andersen, P. & Gholami, R. Mixing dynamics and recovery factor during hydrogen storage in depleted gas reservoirs. *Gas Sci. Eng.* **128**, 205382 (2024).

[2] Flesch, S., Pudlo, D., Albrecht, D., Jacob, A. & Enzmann, F. Hydrogen underground storage—petrographic and petrophysical variations in reservoir sandstones from laboratory experiments under simulated reservoir conditions. *Int. J. Hydrogen Energy* **43**, 20822–20835 (2018).

[3] Rasul, M. G., Hazrat, M. A., Sattar, M. A., Jahirul, M. I. & Shearer, M. J. The future of hydrogen: challenges on production, storage and applications. *Energy Convers. Manag.* **272**, 116326 (2022).

[4] Heinemann, N. et al. Hydrogen storage in saline aquifers: the role of cushion gas for injection and production. *Int. J. Hydrogen Energy* **46**, 39284–39296 (2021).

[5] Ghaedi, M., Andersen, P. Ø. & Gholami, R. Hydrogen diffusion into caprock: a semi-analytical solution and a hydrogen loss criterion. *J. Energy Storage* **64**, 107134 (2023).

[6] Tackie-Otoo, B. N. & Haq, M. B. A comprehensive review on geo-storage of H2 in salt caverns: Prospect and research advances. *Fuel* **356**, 129609 (2024).

[7] Caglayan, D. G. et al. Technical potential of salt caverns for hydrogen storage in Europe. *Int. J. Hydrogen Energy* **45**, 6793–6805 (2020).

[8] Dopffel, N. et al. Microbial hydrogen consumption leads to a significant pH increase under high-saline-conditions: implications for hydrogen storage in salt caverns. *Sci. Rep.* **13**, 1–12 (2023). 37386256 10.1038/s41598-023-37630-y PMC10310820

[9] Zhu, S. et al. Hydrogen loss of salt cavern hydrogen storage. *Renew. Energy* **218**, 119267 (2023).

[10] Derakhshani, R., Lankof, L., GhasemiNejad, A. & Zaresefat, M. Artificial intelligence-driven assessment of salt caverns for underground hydrogen storage in Poland. *Sci. Rep.* **14**, 1–15 (2024). 38902291 10.1038/s41598-024-64020-9 PMC11190257

[11] Bérest, P., Brouard, B. & Durup, J. G. Tightness tests in salt-cavern wells. *Oil Gas Sci. Technol.* **56**, 451–469 (2001).

[12] Tarkowski, R. & Czapowski, G. Salt domes in Poland—potential sites for hydrogen storage in caverns. *Int. J. Hydrogen Energy* **43**, 21414–21427 (2018).

[13] Wang, T. et al. Gas transport model in pore heterogeneous bedded salt rock: implications for tightness evaluation of salt cavern gas storage. *Gas Sci. Eng.* **121**, 205185 (2024).

[14] Chen, X. S., Li, Y. P., Jiang, Y. L., Liu, Y. X. & Zhang, T. Theoretical research on gas seepage in the formations surrounding bedded gas storage salt cavern. *Pet. Sci.* **19**, 1766–1778 (2022).

[15] Liu, W. et al. Research on gas leakage and collapse in the cavern roof of underground natural gas storage in thinly bedded salt rocks. *J. Energy Storage* **31**, 101669 (2020).

[16] AbuAisha, M., Rouabhi, A., Billiotte, J. & Hadj–Hassen, F. Non–isothermal two–phase hydrogen transport in rock salt during cycling in underground caverns. *Int. J. Hydrogen Energy* **46**, 6632–6647 (2021).

[17] Yuan, L., Stanley, A., Dehghanpour, H. & Reed, A. Measurement of Helium diffusion in Lotsberg Salt cores: a proxy to evaluate hydrogen diffusion. *Int. J. Hydrogen Energy*10.1016/J.IJHYDENE.2023.08.003 (2023).

[18] Strauch, B., Pilz, P., Hierold, J. & Zimmer, M. Experimental simulations of hydrogen migration through potential storage rocks. *Int. J. Hydrogen Energy* **48**, 25808–25820 (2023).

[19] Song, R., Song, Y., Liu, J. & Yang, C. Multiscale experimental and numerical study on hydrogen diffusivity in salt rocks and interlayers of salt cavern hydrogen storage. *Int. J. Hydrogen Energy* **79**, 319–334 (2024).

[20] Cui, R., Hassanizadeh, S. M. & Sun, S. Pore-network modeling of flow in shale nanopores: Network structure, flow principles, and computational algorithms. *Earth Sci. Rev.* **234**, 104203 (2022).

[21] Javadpour, F. Nanopores and apparent permeability of gas flow in mudrocks (shales and siltstone). *J. Can. Petrol. Technol.* **48**, 16–21 (2009).

[22] Ryzhkov, I. I. et al. Growth of carbon nanotubes inside porous anodic alumina membranes: Simulation and experiment. *Int. J. Heat. Mass. Transf.* **176**, 121414 (2021).

[23] Darabi, H., Ettehad, A., Javadpour, F. & Sepehrnoori, K. Gas flow in ultra-tight shale strata. *J. Fluid Mech.* **710**, 641–658 (2012).

[24] Singh, H., Javadpour, F., Ettehadtavakkol, A. & Darabi, H. Nonempirical apparent permeability of shale. *SPE Reserv. Eval. Eng.* **17**, 414–424 (2014).

[25] Zhang, W. M. et al. A review on slip models for gas microflows. *Microfluid. Nanofluid.* **13**, 845–882 (2012).

[26] Cai, J. et al. A simple permeability model for shale gas and key insights on relative importance of various transport mechanisms. *Fuel* **252**, 210–219 (2019).

[27] Pang, Y., Fan, D. & Chen, S. A novel approach to predict gas flow in entire Knudsen number regime through nanochannels with various geometries. *SPE J.* **26**, 3265–3284 (2021).

[28] Tison, S. Experimental data and theoretical modeling of gas flows through metal capillary leaks. *Vacuum* **44**, 1171–1175 (1993).

[29] Loyalka, S. K. & Hamoodi, S. A. Poiseuille flow of a rarefied gas in a cylindrical tube: solution of linearized boltzmann equation. *Phys. Fluids Fluid Dyn.* **2**, 2061–2065 (1990).

[30] Alanazi, A. et al. Hydrogen, carbon dioxide, and methane adsorption potential on Jordanian organic-rich source rocks: implications for underground H2 storage and retrieval. *Fuel* **346**, 128362 (2023).

[31] Bruggeman, D. A. G. Berechnung Verschiedener Physikalischer Konstanten Von Heterogenen Substanzen. I. Dielektrizitätskonstanten und Leitfähigkeiten Der Mischkörper Aus Isotropen Substanzen. *Ann. Phys.* **416**, 636–664 (1935).

[32] Ertekin, T., Abou-Kassem, J. & King, G. *Basic Applied Reservoir Simulation* (2001).

[33] Chen, X. et al. Tightness and stability evaluation of salt cavern underground storage with a new fluid–solid coupling seepage model. *J. Pet. Sci. Eng.* **202**, 108475 (2021).

[34] Wei, C., Jafari Raad, S. M., Leonenko, Y. & Hassanzadeh, H. Correlations for prediction of hydrogen gas viscosity and density for production, transportation, storage, and utilization applications. *Int. J. Hydrogen Energy*10.1016/J.IJHYDENE.2023.05.202 (2023).

[35] Leachman, J. W., Jacobsen, R. T., Penoncello, S. G. & Lemmon, E. W. Fundamental equations of state for parahydrogen, normal hydrogen, and orthohydrogen. *J. Phys. Chem. Ref. Data* **38**, 721–748 (2009).

[36] Peng, D. Y. & Robinson, D. B. A new two-constant equation of state. *Ind. Eng. Chem. Fund.* **15**, 59–64 (1976).

[37] Gracki, J. A., Flynn, G. P. & Ross, J. Viscosity of nitrogen, helium, hydrogen, and argon from – 100 to 25°C up to 150–250 atm. *J. Chem. Phys.* **51**, 3856–3863 (1969).

[38] Thoraval, A., Lahaie, F., Brouard, B. & Berest, P. A generic model for predicting long-term behavior of storage salt caverns after their abandonment as an aid to risk assessment. *Int. J. Rock. Mech. Min. Sci.* **77**, 44–59 (2015).

[39] Zhang, Z. et al. Tightness evaluation and countermeasures for hydrogen storage salt cavern contains various lithological interlayers. *J. Energy Storage* **50**, 104454 (2022).

[40] Liu, W. et al. Feasibility evaluation of large-scale underground hydrogen storage in bedded salt rocks of China: a case study in Jiangsu province. *Energy* **198**, 117348 (2020).

[41] Wang, T. et al. Tightness of an underground energy storage salt cavern with adverse geological conditions. *Energy* **238**, 121906 (2022).

[42] Liu, W. et al. Optimization of operating pressure of hydrogen storage salt cavern in bedded salt rock with multi-interlayers. *Int. J. Hydrogen Energy* **58**, 974–986 (2024).

[43] Bernabe, Y. The transport properties of networks of cracks and pores. *J. Geophys. Res. Solid Earth* **100**, 4231–4241 (1995).

[44] Ozarslan, A. Large-scale hydrogen energy storage in salt caverns. *Int. J. Hydrogen Energy* **37**, 14265–14277 (2012).

[45] Friend, D. G., Ely, J. F. & Ingham, H. Thermophysical properties of methane. *J. Phys. Chem. Ref. Data* **18**, 583–638 (1989).

